# TGFβ-Dependent Epithelial–Mesenchymal Plasticity in Immortalized Human Atrial Epicardial Cells: An mRNA Profiling Study

**DOI:** 10.3390/cells15141313

**Published:** 2026-07-22

**Authors:** Katja Nowak, René Schramm, Barbara Kaltschmidt, Christian Kaltschmidt, Cornelius Knabbe, Anna L. Höving

**Affiliations:** 1 Ruhr University Bochum, Heart and Diabetes Center NRW, Institute of Laboratory and Transfusion Medicine, 32545 Bad Oeynhausen, Germany; cknabbe@hdz-nrw.de (C.K.);; 2 University Bielefeld, Faculty of Biology, Department of Cell Biology, 33615 Bielefeld, Germany; c.kaltschmidt@uni-bielefeld.de; 3 Ruhr University Bochum, Heart and Diabetes Center NRW, Department of Thoracic and Cardiovascular Surgery, 32545 Bad Oeynhausen, Germany; rschramm@hdz-nrw.de; 4 University Bielefeld, Faculty of Biology, Molecular Neurobiology, 33615 Bielefeld, Germany; barbara.kaltschmidt@uni-bielefeld.de; 5 University Bielefeld, Medical Faculty OWL, Heart and Diabetes Center NRW, Institute of Laboratory and Transfusion Medicine, 32545 Bad Oeynhausen, Germany

**Keywords:** epicardium-derived cell, epithelial-to-mesenchymal-transition, EMT, heart regeneration, epithelial–mesenchymal plasticity, epicardial activation

## Abstract

The adult mammalian heart exhibits limited regenerative capacity. Although the epicardium contributes to cardiac injury responses and remodeling, expandable adult human in vitro models for investigating epicardial activation and epithelial-to-mesenchymal (EMT)-associated cellular responses remain limited. Here, we isolated human epicardium-derived cells from the adult heart auricle, expressing WT1^+^/MSLN^+^/CRIP1^+^ and generated an expandable immortalized epicardium-derived cell (iEPDC) population, allowing the investigation of intercellular dynamics upon EMT activation. TGFβ signaling was modulated using SB431542 or TGFβ3. Morphological, immunocytochemical, transcriptomic and functional analyses were performed to investigate treatment-dependent responses. SB431542-treated iEPDCs displayed epithelial-like characteristics and elevated WT1, MSLN and CRIP1 expression, with CRIP1 detected at both transcript and protein levels. TGFβ3-treated cells expressed the mesenchymal markers VIM and CD105 and exhibited spindle-shaped morphology, increased migratory behavior and upregulation of mesenchymal- and remodeling-associated markers. Transcriptomic analyses revealed distinct treatment-dependent profiles, enrichment of ‘focal adhesion’, ‘ECM-receptor interaction’ and cytoskeleton-associated pathways in TGFβ3-treated iEPDCs and intermediate transcriptional characteristics in untreated cells. Together, these findings establish adult iEPDCs as an expandable in vitro model for investigating TGFβ-dependent epicardial activation and EMT-associated processes in the adult human heart. Furthermore, the integration of phenotypic, transcriptomic and functional findings revealed the treatment-responsive plasticity of adult human iEPDCs, supporting future studies of injury-associated epicardial activation.

## 1. Introduction

The adult human heart has only a limited capacity to replace myocardium lost after injury [1]. Over the past two decades, studies in zebrafish and mammalian model systems have established the epicardium as an active component of the cardiac injury response [2,3,4]. Following injury, the predominantly quiescent adult epicardium becomes reactivated, although the extent and functional consequences of epicardial activation differ between species, developmental stages and injury models [4].

The epicardium, which forms the outermost layer of the heart, is a sheet composed of mesothelial cells. In the fetal mammalian heart, a subgroup of epicardial cells undergoes epithelial-to-mesenchymal transition (EMT). During EMT, epicardial cells lose epithelial characteristics and acquire migratory mesenchymal-like properties [5,6]. Epicardial cells undergoing EMT migrate through the subepicardial space into the myocardium and are designated epicardium-derived cells (EPDCs) [7]. These EPDCs predominantly differentiate into fibroblasts, smooth muscle cells and pericytes [8,9,10,11]. Whether EPDCs contribute to cardiomyocyte and endothelial lineages remains debated, particularly in the adult mammalian heart [12,13,14].

In experimental models of ischemic heart disease, including myocardial infarction, the epicardium is activated, and the cells begin to proliferate and, in part, migrate into the myocardium, undergoing EMT-associated transitions with tissue remodeling [7,15]. In addition to these processes, epicardial paracrine signaling contributes to intercellular communication in cardiac development and injury-associated remodeling by facilitating cell-to-cell communication and influencing repair mechanisms [16].

Despite increasing interest in epicardial biology, well-characterized and expandable human in vitro systems for investigating adult EPDC activation and EMT-associated processes remain limited [17,18]. In general, primary human cells exhibit limited proliferative capacity and progressively undergo senescence during extended culture, thereby restricting their long-term experimental use [19,20,21]. Similar limitations have been described for primary adult EPDCs, which gradually cease proliferation during prolonged cultivation [17]. Ge and colleagues previously established an inducible proliferative human EPDC model and demonstrated TGFβ-associated EMT using selected molecular and functional readouts [17]. However, treatment-associated phenotypic and transcriptional responses in adult human iEPDC models remain insufficiently characterized, particularly across donor-derived populations and through the combined use of morphological, quantitative immunocytochemical, protein-coding transcriptomic and migration analyses.

Myocardial infarction is accompanied by a complex inflammatory and paracrine microenvironment involving cytokine-mediated processes. In addition to pro-inflammatory cytokines such as TNFα, IL-1β and IL-6, members of the TGFβ family are upregulated in the infarcted myocardium, where they contribute to inflammatory resolution, extracellular matrix (ECM) remodeling and scar formation [22,23]. TGFβ3 has been reported to be increased in human myocardial infarction tissue and associated with post-infarction fibrotic remodeling [23], supporting its relevance in injury-associated cardiac repair processes. TGFβ signaling is considered a central regulator of epicardial activation and EMT in both developmental and injury-associated contexts [24,25,26]. Therefore, TGFβ3 represents a suitable stimulus to investigate EMT-associated activation in human EPDC models. In human EPDCs, TGFβ3 has been shown to induce morphological remodeling, increased migratory behavior and modulation of epithelial- and mesenchymal-associated markers [24,25].

Here, we generated and characterized donor-derived immortalized epicardium-derived cells (iEPDCs) from adult human atrial appendage (auricle) tissue to investigate epicardial activation-associated processes. Using modulation of the TGFβ signaling pathway, we assessed whether the cells retained treatment-responsive epicardial identity and mesenchymal-associated phenotypic plasticity. To extend previous work on expandable human EPDC models, we investigated these responses across donor-derived iEPDC populations and complemented phenotypic characterization with protein-coding transcriptomic analysis of TGFβ-dependent gene expression patterns. We integrated morphological, immunocytochemical, transcriptomic and scratch-wound analyses, including characterization of CRIP1, a recently proposed marker of an epithelial-like epicardial state that has been linked to EMT-associated processes [27]. Together, these complementary analyses characterize TGFβ-dependent phenotypic, transcriptional, and migratory responses in donor-derived adult human iEPDCs.

## 2. Materials and Methods

### 2.1. Isolation and Culture of Primary Adult Human Epicardium-Derived Cells

Human heart auricles were obtained during cardiac surgery, such as aortic valve replacement. Within this study, heart auricles from 5 donors were processed: 3 male donors and 2 female donors with donor ages between 62 and 73 years. According to Dronkers and colleagues, human EPDCs were isolated from the human adult heart auricle tissue [18]. In brief, the epicardium was carefully separated from the underlying myocardium and cut into small pieces. Three rounds of trypsin-EDTA (Sigma-Aldrich, Taufkirchen, Germany) were performed at 37 °C for a total of 30 min. After digestion, the cell suspension was passed through a series of syringes (18G to 20G) and through a 100 µm Falcon cell strainer (Fisherbrand, Thermo Fisher Scientific, Schwerte, Germany). The cells were then plated on dishes coated with 0.1% gelatine A (Sigma-Aldrich, Taufkirchen, Germany) and cultured with EPDC-medium + SB431542, a TGFβRI inhibitor, containing DMEM low-glucose (10567014; Thermo Fisher Scientific, Schwerte, Germany) and M199 (31150022; Thermo Fisher Scientific, Schwerte, Germany) mixed at a ratio of 1:1, supplemented with 10% fetal calf serum (Sigma-Aldrich, Taufkirchen, Germany), 1% penicillin/streptomycin (Sigma-Aldrich, Taufkirchen, Germany) and 10 µM SB431542 (MedChemExpress, Sollentuna, Sweden) to maintain the epithelial-like phenotype. The cells were cultured in a humidified incubator at 37 °C with 5% O_2_ and 5% CO_2_. Every two or three days, the cells were fed with fresh EPDC-medium + SB431542.

For passaging, the culture medium was discarded, and the cells were washed once with 1× phosphate-buffered saline (PBS; PAN Biotech, Aidenbach, Germany). Subsequently, 200 µL of trypsin-EDTA was added per well of a 6-well plate and incubated for 5 min at 37 °C. The detaching process was stopped by adding four volumes of fresh EPDC-medium + SB431542. The resulting cell suspension was used for subsequent experiments.

### 2.2. Lentiviral Transduction of EPDCs

EPDCs were transduced with two lentiviral transfer vectors: pHAGE2-TetOminiCMV-SV40LT construct and FUW-M2rtTA. The following plasmids were obtained from Addgene: pHAGE2-TetOminiCMV-SV40LT was a gift from Hans Schöler (Addgene plasmid # 136616). FUW-M2rtTA was a gift from Rudolf Jaenisch (Addgene plasmid # 20342). Lentiviral particle production was conducted in HEK293-FT cells (Invitrogen, Carlsbad, CA, USA) using a lentiviral packaging system kindly gifted by David Baltimore, including the packaging plasmid Δ8.91 and vesicular stomatitis virus glycoprotein (VSV-G)-mediated pseudotyping, together with one of the two transfer vectors via calcium-phosphate precipitation, as previously described [28]. The HEK293-FT cells were cultured in DMEM (PAN Biotech, Aidenbach, Germany) containing 10% fetal calf serum (FCS; Sigma Aldrich, St. Louis, MO, USA) and 2 mM L-glutamine (Sigma Aldrich, St. Louis, MO, USA). After 24 h, the culture medium was replaced. Supernatants containing lentiviral particles were harvested 53 h after transfection, and the supernatant was filtered through a 0.45 µm PES filter and concentrated using the Lenti-X Concentrator (TakaraBio, Saint-Germain-en-Laye, France). The transduction of EPDCs was facilitated by polybrene (Sigma-Aldrich, St. Louis, MO, USA) at a concentration of 10 µg/mL. A total of 24 h after transduction, the medium was replaced, and doxycycline (D9891, Sigma-Aldrich, St. Louis, MO, USA) was added to a final concentration of 100 ng/mL. Successful transduction was confirmed by immunocytochemistry (ICC) for SV40LT.

As SV40LT expression was detectable independently of doxycycline supplementation after 14 days, all subsequent experiments were performed without doxycycline.

Primary EPDCs at passages 0–1 were used for lentiviral immortalization, whereas iEPDCs at passages 4–14 were used for subsequent experimental analyses.

### 2.3. Immunocytochemical Staining

For treatment experiments, 1 × 10^4^ per well were seeded in 8-well slides (ibidi, cells in focus, Gräfelfing, Germany) coated with 0.1% gelatine-type-A in EPDC-medium + SB431542. After 24 h, the cells were washed once with PBS and treated for 5 days with EPDC medium (10% FCS, DMEM/M199 1:1; penicillin/streptomycin) supplemented with either 10 µM SB431542, 1 ng/mL TGFβ3 (PeproTech, Hamburg, Germany) and the corresponding amount of dimethyl sulfoxide (DMSO) (TGFβ3), or DMSO only (NoAdditive, untreated).

For immunocytochemical staining, the culture medium was removed, and the cells were washed once with PBS before being fixed with 4% paraformaldehyde (PFA) for 15 min at room temperature.

Following three washing steps with PBS, the cells were permeabilized and blocked using 0.02% PBS + Triton X-100 (PBT) containing 5% goat serum (Dianova, Hamburg, Germany) or 5% donkey serum (Biozol, Eching, Germany) for 30 min, depending on which primary and secondary antibody was used. Subsequently, the cells were washed once with PBS. The primary antibodies were applied in the blocking solution for one hour (Table 1). After three rinses with PBS, the secondary antibodies Alexa Fluor 555-conjugated goat anti-rabbit IgG (H + L) (A-21428; Invitrogen, Carlsbad, CA, USA) and Alexa Fluor 488-conjugated goat anti-mouse IgG (H + L) (A-11001, Invitrogen, Carlsbad, CA, USA) or Alexa Fluor 555 donkey anti-goat (A-21432, Invitrogen, Carlsbad, CA, USA) diluted 1:300 in PBS were applied for one hour at room temperature. For Phalloidin (F-actin) staining, Alexa Fluor™ 488 Phalloidin in 1% BSA (8076.2, Carl Roth, Karlsruhe, Germany) in PBS was applied for one hour at RT. After three washing steps with PBS, the cells were mounted using ibidi mounting medium with DAPI (ibidi, cells in focus, Gräfelfing, Germany). Fluorescence imaging was performed by confocal laser scanning microscopy (STELLARIS 8 FALCON, Leica Microsystems, Wetzlar, Germany). For image processing, Leica LAS X (Leica Microsystems, Wetzlar, Germany), ImageJ (version 1.54p; National Institutes of Health, Bethesda, MD, USA) and CorelDRAW 2021 (Corel Corporation, Ottawa, ON, Canada) were used.

ICC fluorescence signals were quantified using ImageJ. For MSLN and CRIP1, fluorescence was quantified as threshold-based integrated density of the respective marker channel due to their punctate staining pattern. Values were normalized to the number of DAPI-positive nuclei and expressed relative to SB431542-treated iEPDCs of the same donor. For WT1, nuclear fluorescence intensity was quantified within DAPI-defined nuclear regions and expressed relative to SB431542-treated iEPDCs from the same donor. Due to their filamentous and more diffuse staining pattern, VIM and CD105 were quantified as mean fluorescence intensity of threshold-defined positive regions, normalized to DAPI-positive nuclei and expressed relative to SB431542-treated iEPDCs from the same donor. Image processing and statistical analysis were performed using ImageJ (version 1.54p; National Institutes of Health, Bethesda, MD, USA), CorelDRAW 2021 (Corel Corporation, Ottawa, ON, Canada), and GraphPad Prism 8.3.0 (GraphPad Software, Inc., Boston, MA, USA).

### 2.4. RNA Sequencing

For RNA sequencing, 1 × 10^5^ cells per well were seeded in 0.1% gelatine-type-A-coated 6-well plate (Sarstedt AG and Co., Nümbrecht, Germany) in EPDC medium + SB431542 and cultivated for 24 h. After 24 h, the cells were washed with PBS and treated for five days with EPDC medium (10% FCS, DMEM/M199 1:1; penicillin/streptomycin) supplemented with either 10 µM SB431542, 1 ng/mL TGFβ3 and the corresponding amount of DMSO (TGFβ3), or DMSO only (NoAdditive). RNA was isolated using the NucleoSpin RNA Kit (Macherey-Nagel GmbH & Co. KG, Düren, Germany) according to the manufacturer’s guidelines. The concentration of the isolated RNA was determined using a NanoDrop spectrophotometer (Peqlab, Erlangen, Germany). Subsequently, the samples were stored at −80 °C. RNA was sequenced by Novogene (Munich, Germany) using the Illumina NovaSeq X Plus Series platform with a paired-end 150 bp strategy. Additional RNAseq datasets were obtained from the NCBI Sequence Read Archive under the accession numbers: GSE129547 (human cardiac stem cells, hCSCs) [29] and GSE301005 (human pluripotent stem cell-derived epicardial cells, hPSC-EPC). Both reference datasets were generated using Illumina paired-end sequencing, ensuring technical compatibility with the iEPDC dataset. From the latter study, only the control group dataset was selected to include expression data from untreated cells.

Initially, all data underwent the same processing procedure: a primary quality control of the raw data was conducted using FastQC (version 0.11.9). Following this, low-quality bases were trimmed, and adapter clipping was performed with Trimmomatic-0.39 [30]. The following settings were applied: PE; -phred33; ILLUMINACLIP:TruSeq3-PE.fa:2:30:10; LEADING:6; TRAILING:6; SLIDINGWINDOW:4:15; MINLEN:36. The alignment of clean reads to the Homo sapiens reference genome sequence (GRCh38.p14) was conducted utilizing HiSat2 [version 2.1.0]. For quantifying the read numbers, after mapping, featurecounts (version 2.0.0) was used with the subsequent parameters: -T 4; -t gene; -g gene_id; -a Homo_sapiens.GRCh38.78.gtf. Transcripts per million (TPM) values were calculated from raw counts generated by featurecounts, normalizing counts to gene length to obtain reads per kilobase and subsequently scaling each sample to a total of 1 × 10^6^ transcripts. Differential gene expression analysis between two groups was performed using the DESeq2 R package [version 1.48.2]. Gene ontology (GO)-term enrichment and Kyoto Encyclopedia of Genes and Genomes (KEGG) pathway analysis were performed by using the clusterProfiler (version 4.16.0) package in R. Information on protein function was obtained from the UniProt database (www.uniprot.org).

### 2.5. Scratch-Assay

For the scratch assay, 1 × 10^5^ cells per well were seeded in 0.1% gelatine-type-A-coated 6-well plate (Sarstedt AG and Co., Nümbrecht, Germany) in EPDC-medium supplemented with SB431542. After 24 h, the cells were washed with PBS and cultured for five days with EPDC medium (10% FCS, DMEM/M199 1:1; penicillin/streptomycin), supplemented either with 10 µM SB431542, with 1 ng/mL TGFβ3 and the equivalent amount of DMSO (TGFβ3), or DMSO alone (NoAdditive). Following the treatment period, a scratch was performed using a 200 µL pipette tip, and the medium was replaced. Images were acquired immediately after scratching (0 h) and after 8 h with a Leica MateoTL (Leica Microsystems, Wetzlar, Germany). The scratch width was calculated with the updated wound healing tool in ImageJ [31]. Image processing and statistical analysis were performed using ImageJ (version 1.54p; National Institutes of Health, Bethesda, MD, USA), CorelDRAW 2021 (Corel Corporation, Ottawa, ON, Canada), and GraphPad Prism 8.3.0 (GraphPad Software, Inc., Boston, MA, USA).

## 3. Results

### 3.1. Preservation of the Primary Marker Profile in Immortalized Epicardium-Derived Cells from the Human Heart Auricle

**Figure 1:**
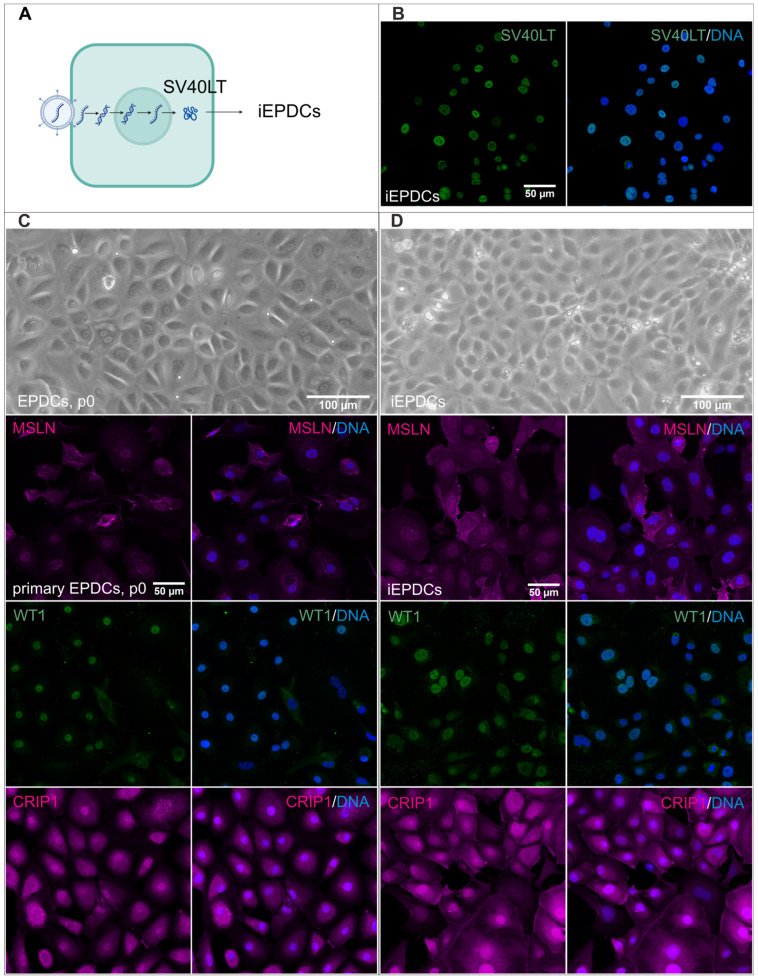
Generation and characterization of immortalized human primary EPDCs. (**A**) Schematic overview of the immortalization strategy. Primary human EPDCs were immortalized by lentiviral transduction with SV40LT. (**B**) Successful immortalization was confirmed by ICC of SV40LT expression in transduced cells. (**C**) Representative brightfield image and ICC marker profile of primary human EPDCs showing expression of MSLN, WT1 and CRIP1. (**D**) Representative brightfield image and ICC marker profile of immortalized human EPDCs showing preserved expression of MSLN, WT1 and CRIP1. (**B**–**D**) Nuclei were counterstained with DAPI.

Primary adult human EPDCs were isolated from the human heart auricle and characterized for the expression of epicardial and EPDC-associated markers. During initial culture, these primary EPDCs exhibited limited proliferative capacity and ceased proliferation, which is consistent with the primary somatic origin. To overcome this limitation and establish an expandable cell source for downstream studies, the EPDCs were immortalized by lentiviral transduction with SV40LT, as schematically illustrated (Figure 1A). Successful immortalization was confirmed by ICC detection of SV40LT in transduced iEPDCs (Figure 1B).

A representative brightfield image and analysis of the ICC marker profile of primary human EPDCs revealed an epithelial, cobblestone-like appearance and expression of MSLN, an epicardium-related marker [32,33,34], WT1, an epithelial-EPDC-associated marker [17,18,32,33] and CRIP1, a proposed epithelial epicardial state marker associated with EMT regulation [27] (Figure 1C). These resulting iEPDCs retained this cobblestone-like morphology and displayed a marker profile comparable to that of the primary EPDCs, including MSLN, WT1 and CRIP1. Even after extended culture (beyond passage 10), this marker profile was largely preserved (Figure S1A).

To assess the doxycycline responsiveness of the inducible expression system, iEPDCs were cultured for 14 days with or without doxycycline. ICC staining showed detectable SV40LT expression under both conditions (Figure S1B). The mean population doubling time of iEPDCs derived from three independent donors was 26.3 ± 4.8 h (Figure S1C).

### 3.2. TGFβ3 Induces Cytoskeletal Remodeling and a Mesenchymal-like Morphology in iEPDCs

To examine how modulation of TGFβ3 signaling affects the morphology of iEPDCs, F-actin organization was analyzed by phalloidin staining.

**Figure 2:**
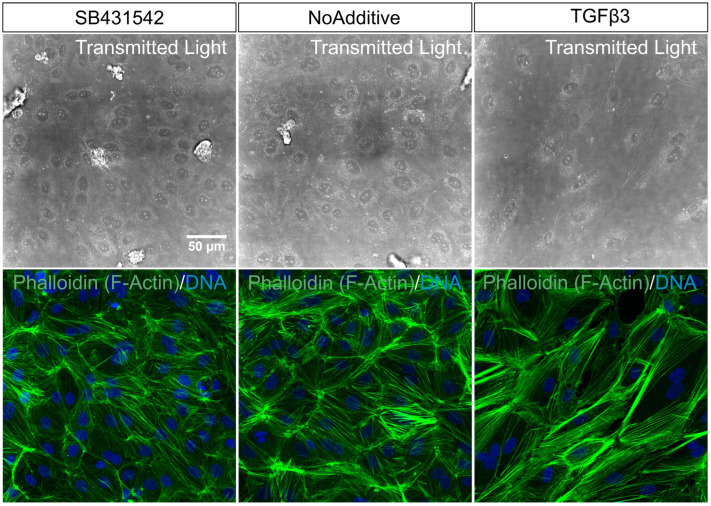
Morphological characterization of iEPDCs after five days of culture with SB431542, NoAdditive and TGFβ3. Phalloidin-F-actin staining illustrated distinct morphological phenotypes of iEPDCs following treatment. TGFβ3-treated iEPDCs exhibited an elongated spindle-shaped morphology, whereas SB431542-treated cells displayed a cobblestone-like appearance. Cells cultured without any additive showed an intermediate morphology with features of both TGFβ3- and SB431542-treated phenotypes. Nuclei were counterstained with DAPI.

Following TGFβ3 treatment, iEPDCs adopted an elongated, spindle-shaped form, indicative of a mesenchymal-like phenotype. In contrast, inhibition of TGFβRI signaling by SB431542 resulted in a cobblestone-like appearance, characteristic of epithelial-like morphology. In the absence of any additive (NoAdditive), iEPDCs displayed an intermediate morphology characterized by features of both the TGFβ3- and SB431542-treated phenotypes (Figure 2).

### 3.3. TGFβ3 Promotes a Mesenchymal-like Marker Profile, While SB431542 Maintains Epithelial-like Characteristics in iEPDCs

**Figure 3:**
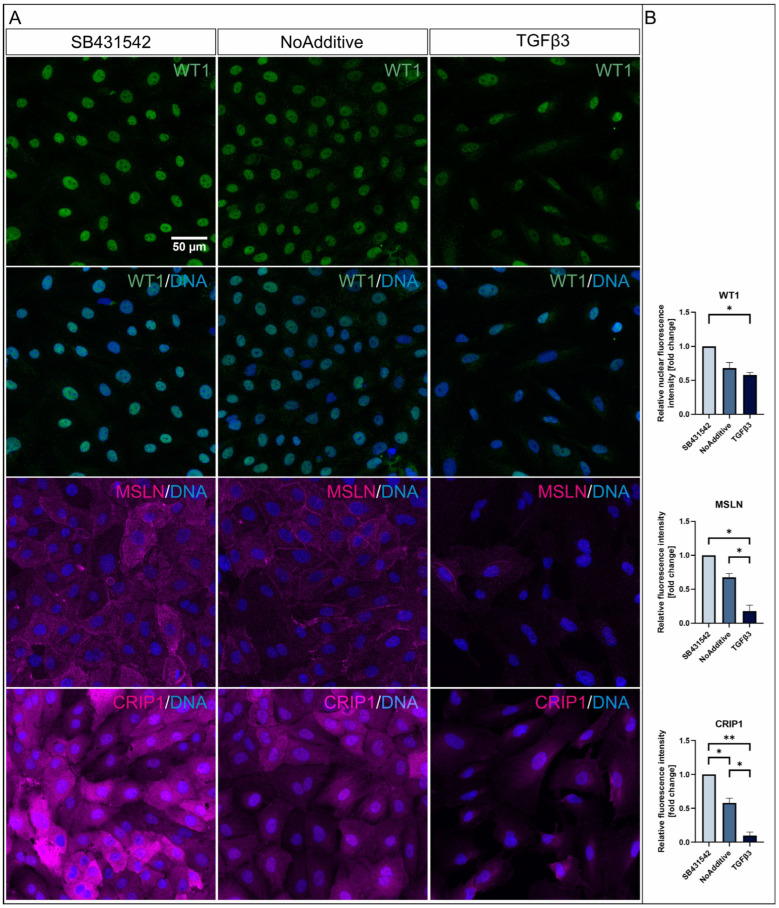
TGFβ3 treatment reduces epithelial-like features in iEPDCs. iEPDCs were cultured for five days under SB431542, NoAdditive or TGFβ3 conditions. (**A**) Immunocytochemical staining of iEPDCs demonstrates lower expression of epithelial-associated markers MSLN and CRIP1 in iEPDCs treated with TGFβ3 for five days compared with SB431542-treated cells, indicating a loss of epithelial characteristics. iEPDC cultures under the NoAdditive condition show intermediate levels of these markers, resembling an expression pattern between SB431542- and TGFβ3-treated cells. Nuclei were counterstained with DAPI. (**B**) Quantification of nuclear WT1 relative fluorescence intensity confirms significantly lower levels of WT1 in TGFβ3-treated iEPDCs compared to SB431542-treated cells. Relative fluorescence intensities of MSLN and CRIP1, determined by threshold-based analysis, were similarly decreased following TGFβ3 treatment compared to SB431542-treated iEPDCs. All data are shown as mean ± SEM, RM one-way ANOVA with Tukey’s post hoc test; *p* * < 0.05; *p* ** < 0.01; *n* = 3 donors.

To characterize the epithelial-like phenotype in iEPDCs, the expression of epithelial-associated markers MSLN, CRIP1, and WT1 was analyzed. Expression of nuclear WT1 was significantly lower in TGFβ3-treated iEPDCs compared to SB431542-treated cells (Figure 3A,B). MSLN expression was significantly reduced in TGFβ3-treated iEPDCs compared to both SB431542- and NoAdditive-treated cells, while no significant difference was observed between the SB431542 and NoAdditive conditions (Figure 3A,B). Similarly, CRIP1 expression was lowest in TGFβ3-treated iEPDCs. SB431542-treated cells showed significantly higher CRIP1 expression than NoAdditive-treated cells, whereas TGFβ3-treated cells differed significantly from both conditions (Figure 3A,B). Further, NoAdditive-iEPDCs displayed expression levels of epithelial-associated markers that were higher than in TGFβ3-treated cells and similar to SB431542-treated cells (Figure 3A). These results indicate a loss of epithelial-like characteristics in response to TGFβ3 in iEPDCs, whereas NoAdditive-treated iEPDCs retained selected epithelial-associated features.

**Figure 4:**
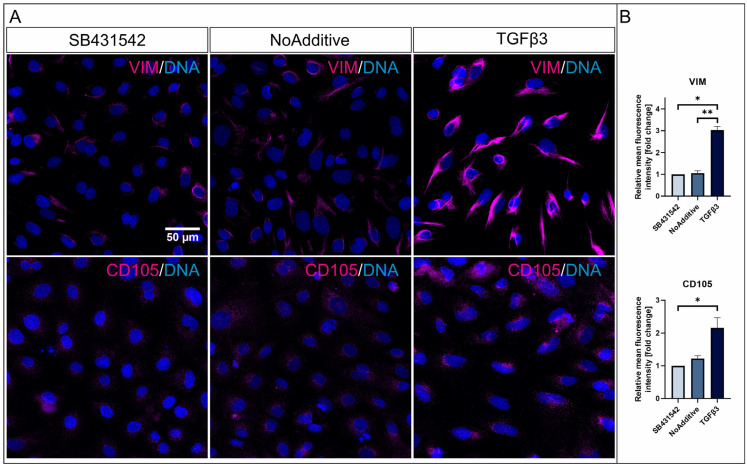
TGFβ3 enhances mesenchymal-like features in iEPDCs. iEPDCs were cultured for five days under SB431542, NoAdditive or TGFβ3 conditions. (**A**) Immunocytochemical staining of iEPDCs shows that VIM and CD105/Endoglin expression levels are elevated in iEPDCs treated with TGFβ3 compared with SB431542-treated cells. iEPDCs cultured with NoAdditive show intermediate levels of both markers. The increased abundance and filamentous organization of VIM in TGFβ3-treated iEPDCs reflect cytoskeletal remodeling associated with a mesenchymal-like phenotype. Nuclei were counterstained with DAPI. (**B**) Quantification of VIM and CD105 relative mean fluorescence intensity confirms higher levels in TGFβ3-treated iEPDCs compared to SB431542-treated cells. All data are shown as mean ± SEM, RM one-way ANOVA with Tukey’s post hoc test; *p* * < 0.05; *p* ** < 0.01; *n* = 3 donors.

To examine the mesenchymal-like characteristics in iEPDCs, the expression of mesenchymal-associated markers VIM and CD105/Endoglin were analyzed. Consistent with the morphological shift toward a spindle-shaped appearance (Figure 2), TGFβ3-treated iEPDCs showed increased expression of these mesenchymal-associated markers VIM and CD105 compared with SB431542-treated cells (Figure 4A,B). Further, iEPDCs cultured with NoAdditive displayed intermediate expression levels of VIM and CD105, lower than in TGFβ3-treated but higher in SB431542-treated cells. Moreover, the VIM intermediate filaments appeared more elongated in TGFβ3-treated iEPDCs, reflecting cytoskeletal remodeling associated with a mesenchymal-like state. These findings support the notion that TGFβ3 promotes the acquisition of mesenchymal-like features in iEPDCs.

### 3.4. RNA Sequencing Reveals a Transcriptional Profile of iEPDCs with Shared Features of Human Cardiac Stem Cells and hPSC-Derived Epicardial Cells

To characterize the transcriptional profile of iEPDCs from the human atrial appendage treated with SB431542, NoAdditive and TGFβ3, we compared RNAseq data of iEPDCs with human cardiac stem cells (hCSCs; GSE129547) from the same region and human pluripotent stem cell-derived epicardial cells (hPSC-EPCs; GSE301005). As primary cardiac cell control, hCSCs were included, and hPSC-EPCs represented in vitro-generated epicardial-like cells. These datasets were chosen because they were generated using Illumina paired-end sequencing, matching the iEPDCs dataset, which ensured technical comparability. Principal component analysis (PCA) revealed that all cell populations formed distinct clusters.

**Figure 5:**
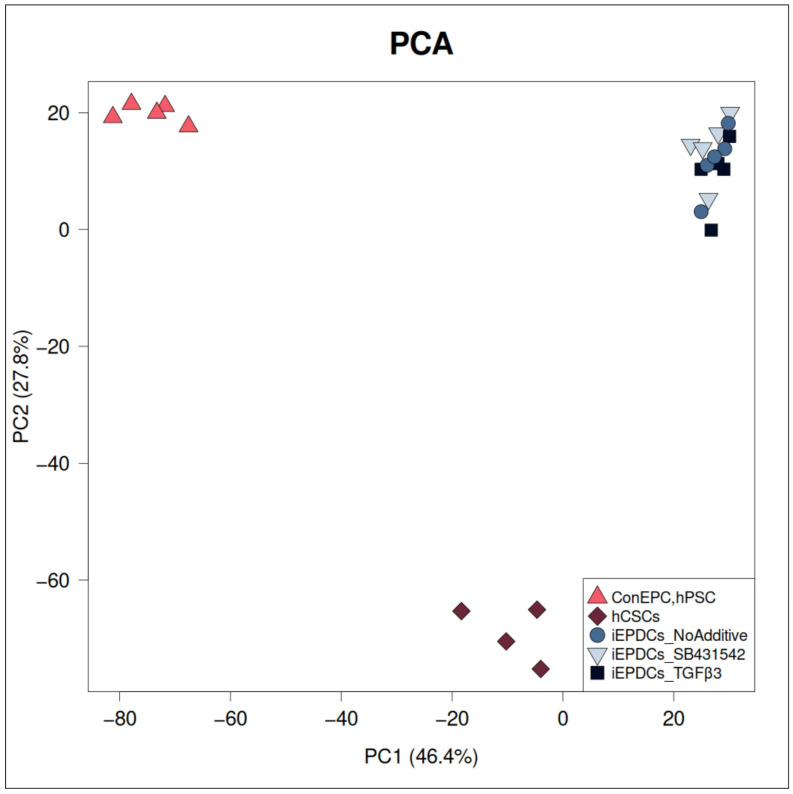
Principal component analysis (PCA) of iEPDCs and related cell populations. PCA of iEPDCs cultured for five days under SB431542-, NoAdditive- or TGFβ3-conditions, together with hCSCs and hPSC-derived epicardial cells, reveals that all cell populations cluster independently.

iEPDCs and hPSC-EPCs aligned horizontally and showed similar PC2 values, with most variation explained by PC1, indicating a transcriptional similarity between iEPDCs and hPSC-EPCs (Figure 5). In contrast, primary hCSCs from the human myocardium aligned vertically relative to iEPDCs, displaying comparable PC1 values, but with most variation explained by PC2 (Figure 5). These findings indicate partial transcriptional similarity of iEPDCs to both primary hCSCs and hPSC-derived epicardial cells, while supporting their distinction as a separate cell population.

To compare the markers assessed on protein level with their corresponding transcript levels, TPM values for *WT1*, *MSLN*, *CRIP1*, *VIM* and *ENG/CD105* were examined descriptively in the bulk RNAseq dataset of iEPDCs (Figure S2). The clearest treatment-associated pattern was observed for *CRIP1*, with higher transcript abundance in SB431542-treated iEPDCs and lower abundance following TGFβ3 treatment. *MSLN* showed a similar tendency, although the magnitude of the response varied between donors. *WT1* showed only minor differences between conditions, whereas *VIM* and *ENG* tended to be higher in NoAdditive- and TGFβ3-treated iEPDCs, accompanied by donor-dependent variability.

To extend this marker-based comparison, additional genes associated with EMT, ECM remodeling and cellular identity were examined. TGFβ3-treated iEPDCs tended to show higher abundance of the EMT-associated factors *SNAI1*, *SNAI2*, and *TWIST1*, as well as *CDH2* and the matrix-remodeling genes *MMP2* and *MMP9* (Figure S3A). *GATA4, TCF21*, *TBX18* and *TNNT2* were additionally examined to further characterize the cellular identity of the iEPDCs (Figure S3B).

### 3.5. RNA Sequencing Reveals Epithelial- and Mesenchymal-Associated Transcriptional Programs in iEPDCs

To comprehensively characterize treatment-induced transcriptional changes, SB431542-, NoAdditive- and TGFβ3-treated iEPDCs were analyzed by RNAseq. Differentially expressed genes (DEGs) identified under each condition were systematically compared to assess treatment-specific transcriptional responses.

**Figure 6:**
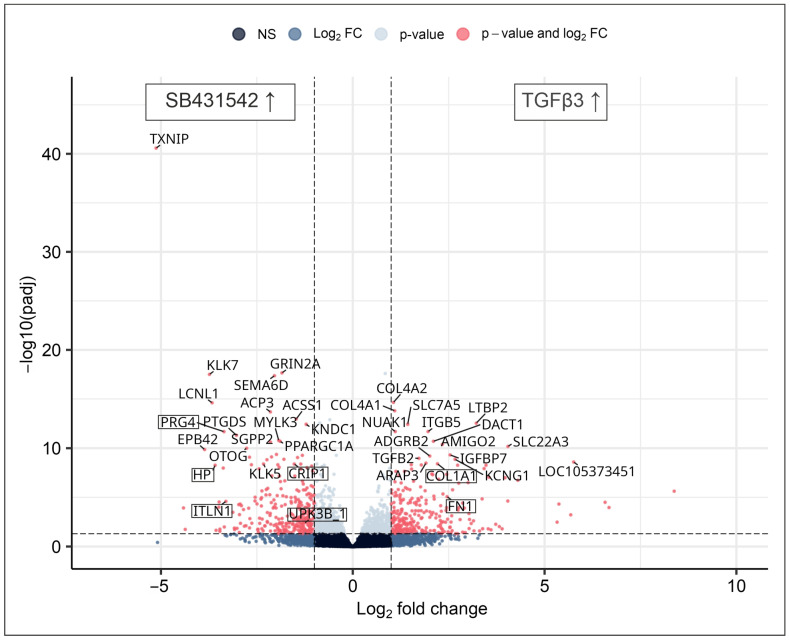
Differential gene expression in TGFβ3- versus SB431542-treated iEPDCs after five days of treatment. Volcano plot of differentially expressed genes shows upregulation of 287 genes in TGFβ3-treated iEPDCs and upregulation of 293 genes in SB431542-treated iEPDCs. The boxes highlight genes associated with EPDCs and the epicardium. The complete list of DEGs is provided in Supplementary Table S1.

Comparison of TGFβ3-treated and SB431542-treated iEPDCs revealed a total of 580 significantly differentially expressed genes, including 287 genes upregulated in TGFβ3-treated iEPDCs and 293 genes upregulated in SB431542-treated iEPDCs (Figure 6). *ITLN1*, *MSLN*, *UPK3B*, *HP*, *PRG4* and *CRIP1* were upregulated in SB431542-treated iEPDCs compared to TGFβ3-treated cells. These genes have been associated with mesothelial or epicardial cell populations in scRNAseq of human fetal and adult epicardium [27,32], supporting maintenance of an epithelial-like epicardial transcriptional profile under SB431542 treatment. Furthermore, genes upregulated in SB431542-treated iEPDCs were *TXNIP*, *PTGDS* and various metallothioneins (Supplementary Table S1). In contrast, *COL1A1* and *FN1* were upregulated in TGFβ3-treated iEPDCs compared to SB431542-treated cells. Both genes have previously been reported to be induced in TGFβ3-treated primary and inducible proliferative EPDCs [17], consistent with the transcriptional changes observed in iEPDCs. Furthermore, *ATF6B*, *TGFB2* and *LTBP2* were upregulated in TGFβ3-treated iEPDCs compared to SB431542-treated cells.

**Figure A1:**
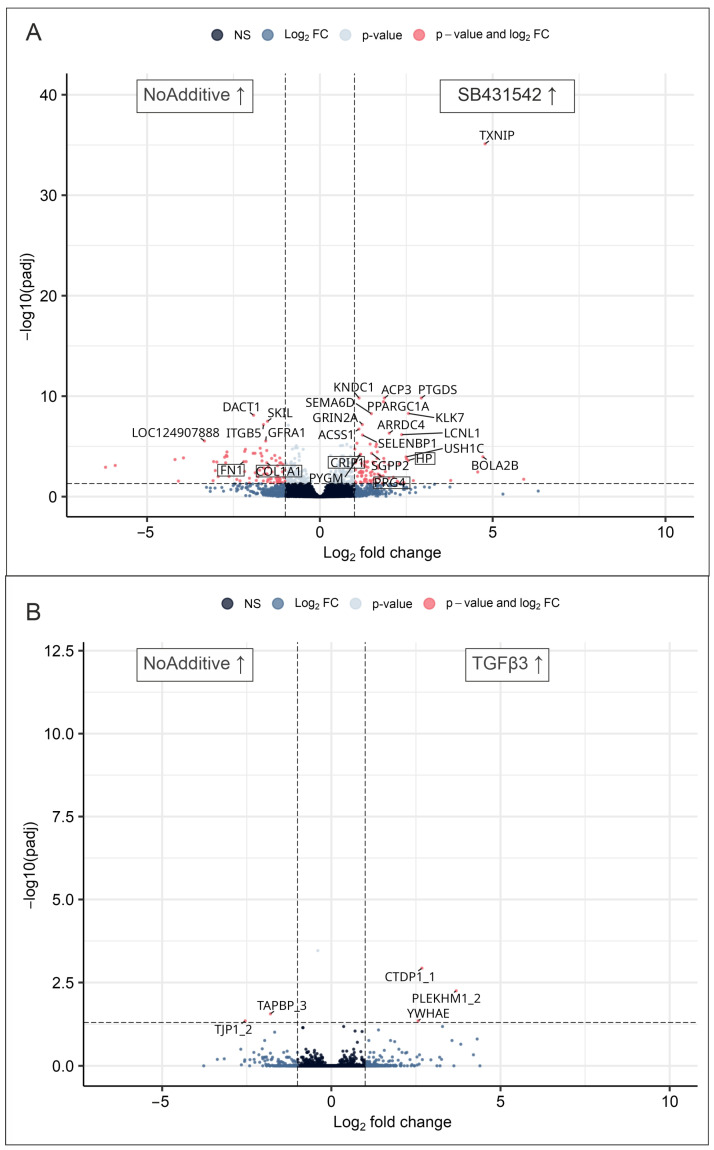
(**A**) Volcano plot of differentially expressed genes in EPDCs treated with SB431542 vs. NoAdditive. (**B**) Volcano plot of differentially expressed genes in EPDCs treated with TGFβ3 vs. NoAdditive. The boxes highlight genes associated with EPDCs and the epicardium. Volcano plot showing DEGs after 5 days of treatment. Complete differential expression results for the respective comparisons are provided in Supplementary Tables S2 and S3.

In the comparison between SB431542- and NoAdditive-treated iEPDCs, a total of 176 genes were significantly differentially expressed, with 92 genes upregulated in SB431542-treated iEPDCs and 84 genes upregulated in NoAdditive-treated iEPDCs (Figure A1A). Among the genes upregulated in SB431542-treated cells were *HP*, *CRIP1* and *PRG4*.

When comparing NoAdditive- and TGFβ3-treated iEPDCs, six genes were identified as differentially expressed, including four genes upregulated in TGFβ3-treated iEPDCs and two genes upregulated in NoAdditive-treated iEPDCs (Figure A1B). The comparatively small number of DEGs between these two conditions indicates a closer transcriptional resemblance of NoAdditive-treated iEPDCs to the TGFβ3-induced state than to SB431542-treated cells. The complete lists of DEGs for all pairwise comparisons are provided in Supplementary Tables S1–S3.

### 3.6. RNA Sequencing Reveals Treatment-Dependent Pathway Enrichment in iEPDCs

**Figure 7:**
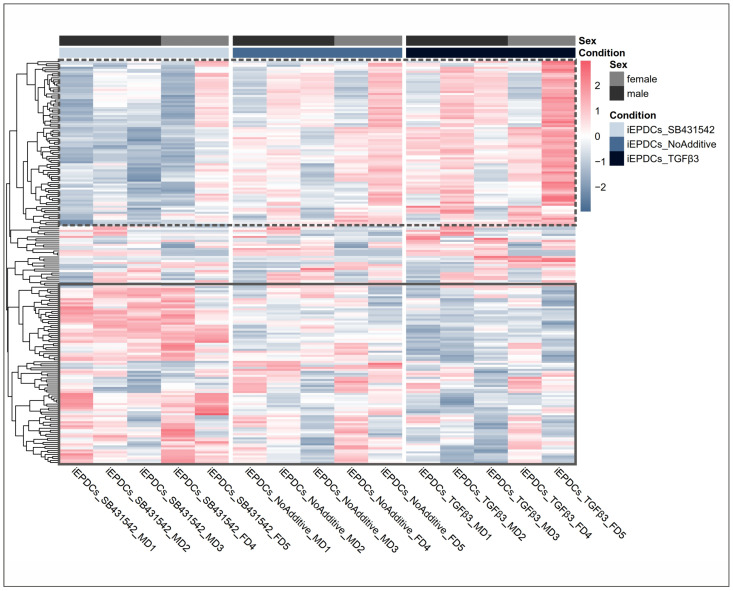
Heatmap of the top 200 differentially expressed genes in iEPDCs treated with SB431542, NoAdditive and TGFβ3 for five days. The dashed box indicates genes that are mainly upregulated in TGFβ3-treated iEPDCs and downregulated under SB431542 conditions, whereas the solid box highlights genes that are mainly upregulated in SB431542-treated iEPDCs and mainly downregulated in TGFβ3-treated cells. A version of the heatmap displaying all gene labels is provided in Supplementary Figure S4.

The heatmap visualization of RNAseq-derived DEGs of iEPDCs under the three treatments SB431542, NoAdditive and TGFβ3 revealed two major clusters (Figure 7). The upper cluster (dashed box) comprised genes that were downregulated in SB431542-treated iEPDCs and upregulated in NoAdditive- and TGFβ3-treated iEPDCs. The lower cluster (solid box) contained genes that were upregulated in SB431542-treated cells and downregulated in NoAdditive- and TGFβ3-treated iEPDCs. Notably, NoAdditive-iEPDCs displayed an intermediate transcriptional pattern between SB431542- and TGFβ3-treated cells. These data are consistent with a gradual shift in transcriptional state rather than a strictly binary response.

**Figure 8:**
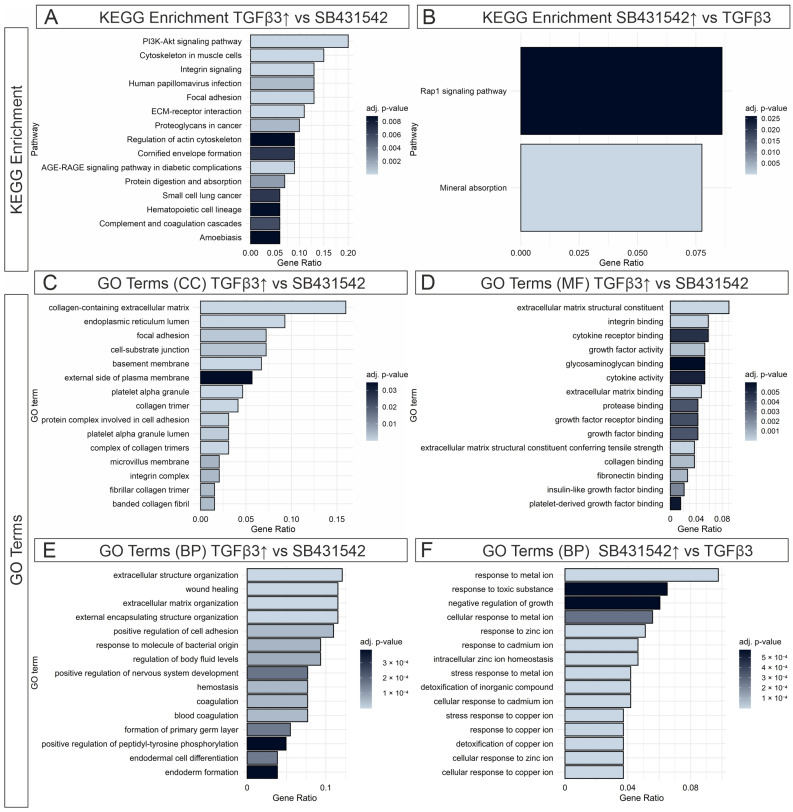
KEGG and GO enrichment analyses of TGFβ3-treated vs. SB431542-treated iEPDCs after five days of treatment. (**A**) Upregulated KEGG terms in TGFβ3-treated iEPDCs compared with SB431542-treated cells. (**B**) Upregulated KEGG terms in SB431542-treated iEPDCs compared with TGFβ3-treated cells. Upregulated GO terms in TGFβ3-treated iEPDCs compared with SB431542-treated in (**C**) cellular component, (**D**) molecular function and (**E**) biological process. (**F**) Upregulated biological process GO terms in SB431542-treated iEPDCs compared with TGFβ3-treated cells. For each analysis, the top 15 significantly enriched pathways/terms are displayed.

Kyoto Encyclopedia of Genes and Genomes (KEGG) pathway analysis revealed significant enrichment of ‘PI3K-Akt signaling pathway’ in TGFβ3-treated iEPDCs compared to SB431542-treated iEPDCs (Figure 8A). Pathways associated with cell–matrix interactions, including ‘integrin signaling’, ‘focal adhesion’ and ‘ECM-receptor interaction’ were upregulated in TGFβ3-treated iEPDCs compared to SB431542-treated cells. Additionally, cytoskeleton-associated pathways were enriched in TGFβ3-treated iEPDCs compared to SB431542-treated cells: ‘Cytoskeleton in muscle cells’ and ‘Regulation of actin cytoskeleton’. In contrast, ‘Rap1 signaling pathway’ was upregulated in SB431542-treated iEPDCs compared to TGFβ3-treated cells (Figure 8B).

Gene Ontology (GO) enrichment analysis further supported the activation of mesenchymal-associated programs in TGFβ3-treated iEPDCs compared to SB431542-treated cells. In the cellular component category, enriched terms included ‘collagen-containing extracellular matrix’, ‘protein complex involved in cell adhesion’ and ‘focal adhesion’, indicating remodeling of extracellular and adhesion-associated structures (Figure 8C). In the molecular function category, significantly enriched terms comprised ‘extracellular matrix structural constituent’, ‘integrin binding’, ‘extracellular matrix binding’, ‘protease binding’, ‘extracellular matrix structural constituent conferring tensile strength’, ‘collagen binding’ and ‘fibronectin binding’, consistent with enhanced matrix interaction and structural ECM organization (Figure 8D). Within the biological process category, enriched terms included ‘extracellular structure organization’, ‘wound healing’, ‘extracellular matrix organization’ and ‘positive regulation of cell adhesion’, reflecting activation of tissue remodeling and migration-associated processes (Figure 8E).

### 3.7. TGFβ3 Enhances the Migratory Capacity of iEPDCs Compared to SB431542 Treatment

**Figure 9:**
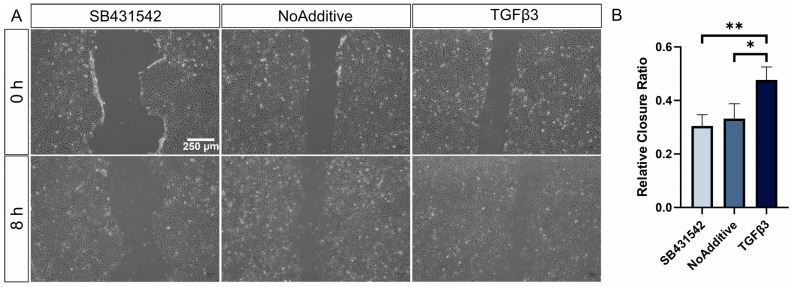
TGFβ3-treated mesenchymal-like iEPDCs show a higher relative closure ratio than SB431542-treated cells after 8 h. iEPDCs were cultured for five days under SB431542, NoAdditive or TGFβ3 conditions before scratching. (**A**) Representative images of a scratch after 0 h and 8 h. (**B**) Relative closure ratio indicates a higher migration rate of TGFβ3-treated iEPDCs compared to SB431542-treated cells. Data are shown as mean ± SEM, RM one-way ANOVA with Tukey’s post hoc test; *p* * < 0.05; *p* ** < 0.01; *n* = 5 donors.

To compare the migratory capacity of SB431542-treated, NoAdditive- and TGFβ3-treated iEPDCs, a scratch assay was performed. All iEPDCs migrated into the cell-free zone and contributed to closure of the gap (Figure 9A). The relative gap closure ratio indicated that TGFβ3-treated iEPDCs migrated significantly faster than SB431542-treated iEPDCs after 8 h (Figure 9B).

## 4. Discussion

Given the limited regenerative capacity of the adult mammalian heart, understanding cellular responses to cardiac injury remains important [1]. Studies using zebrafish and mouse injury models have established the epicardium as an active component of the cardiac injury response, although the extent and functional consequences of epicardial activation differ between species and injury models [2,3,4]. These model-dependent differences underscore the value of human EPDC systems as a complementary platform. Primary fetal and adult human EPDC cultures, as well as an inducible proliferative adult human EPDC model, have previously been established to investigate epicardial activation and EMT-associated processes in vitro [17,18,24,25]. However, primary adult human EPDCs isolated from the heart auricle exhibit limited proliferative capacity, consistent with their somatic origin and comparable to other primary cell types such as fibroblasts and mesenchymal stem cells [17,19,20,21]. To overcome this limitation and generate an expandable cell source for downstream applications, EPDCs were immortalized using a lentiviral SV40LT expression system.

The resulting immortalized EPDCs displayed an epithelial, cobblestone-like morphology even during extended culture and exhibited a mean population doubling time of 26.3 ± 4.8 h, whereas primary adult human EPDCs have been reported to lose their cobblestone-like morphology and show reduced proliferative capacity as early as population doubling 5 [17]. At the protein level, iEPDCs exhibited retained expression of epicardial and epithelial-associated EPDC markers, including MSLN, WT1 and CRIP1 [17,18,27,32,33,34]. These findings suggest that iEPDCs retained selected epicardial- and epithelial-associated morphological and molecular features following immortalization. Although SV40LT-mediated immortalization may influence cell cycle regulation and proliferation-associated pathways, the preserved epithelial morphology and epithelial and epicardial marker profile support the use of iEPDCs as a complementary in vitro model for investigating EPDC-associated processes.

In the physiological context of cardiac development, epicardial cells undergo EMT and form EPDCs, whereas injury-induced activation of the adult epicardium, including following myocardial infarction, can involve EMT-associated cellular transitions in animal models [3,7,16,35]. TGFβ3 has been implicated in EMT-associated activation of human EPDCs, including morphological remodeling and modulation of epithelial- and mesenchymal-associated markers [17,24,25].

To determine whether iEPDCs retained responsiveness to modulation of TGFβ signaling, we analyzed cell morphology, actin cytoskeleton organization, and epithelial and epicardial marker expression under conditions of TGFβ pathway inhibition (SB431542), NoAdditive and TGFβ3 stimulation. Phalloidin staining showed a cobblestone-like morphology in SB431542-treated iEPDCs and a more elongated, spindle-shaped appearance following TGFβ3-treatment, whereas NoAdditive-treated cells showed an intermediate morphology. Under SB431542 treatment, iEPDCs maintained expression of MSLN, CRIP1, and nuclear WT1, consistent with a more epithelial-like epicardial state. In contrast, TGFβ3-treated iEPDCs exhibited significantly reduced expression of MSLN, CRIP1 and nuclear WT1 compared to SB431542-treated cells. These findings indicate that iEPDCs remained phenotypically responsive to TGFβ3 and reproduced aspects of EMT-associated marker regulation.

To further investigate the mesenchymal-like shift, CD105 and VIM were analyzed at the protein level. TGFβ3-treated iEPDCs showed increased CD105 expression compared with SB431542-treated cells. CD105 (endoglin) is a marker of mesenchymal stem cells and endothelial cells and acts as a coreceptor for TGFβ superfamily proteins [36]. In addition, CD105-positive cell populations have been described in hCSCs [37]. Enhanced *endoglin* expression has been reported following EMT induction in human EPDCs derived from explant culture [38], and our findings support its regulation in response to TGFβ3 in adult human iEPDCs.

To further substantiate the mesenchymal-like characteristics of TGFβ3-treated iEPDCs, VIM was analyzed as an additional mesenchymal marker [39,40]. TGFβ3-treated iEPDCs exhibited elevated VIM expression compared with SB431542- and NoAdditive-treated cells. This was accompanied by a more pronounced filamentous VIM organization in spindle-shaped cells, indicating cytoskeletal remodeling associated with the mesenchymal-like state. In epicardioids, VIM-positive mesenchymal cells have been described underneath a KRT18^+^ epithelial cell layer [41], supporting the relevance of VIM as a marker of mesenchymal-like EPDCs. Notably, VIM was also detectable in a subset of SB431542-treated iEPDCs, although at lower levels than in TGFβ3-treated cells, suggesting that EMT-associated marker expression changes in iEPDCs are not strictly binary.

To further characterize the transcriptional identity of iEPDCs, transcriptomic profiles were compared with previously published datasets. PCA was conducted, including hCSCs, iEPDCs and hPSC-derived epicardial cells. PCA revealed distinct clustering of each cell type, indicating clear differences in overall gene expression profiles. Notably, the two primary tissue-derived cell populations, hCSCs and iEPDCs, clustered closely along PC1, suggesting that their shared origin from human heart auricle tissue may contribute to the observed transcriptional variance. In contrast, hPSC-derived epicardial cells were separated along PC1, while partially aligning with iEPDCs along PC2. Since incomplete maturation has been described in various iPSC-derived cardiac cell populations [42,43], this separation may reflect developmental immaturity and differences in cellular origin. Together, these findings suggest that the transcriptional positioning of iEPDCs relative to hCSCs may in part reflect their shared origin from adult human heart auricle tissue while also indicating similarities to hPSC-derived epicardial cells.

To comprehensively characterize treatment-induced transcriptional changes, SB431542-, NoAdditive- and TGFβ3-treated iEPDCs were analyzed by RNAseq. DEGs identified under each condition were systematically compared to assess treatment-specific transcriptional responses.

Differential gene expression analysis revealed distinct transcriptional profiles between SB431542- and TGFβ3-treated iEPDCs, with 580 DEGs identified between both conditions. In comparison, 176 DEGs were detected between SB431542- and NoAdditive-treated iEPDCs, whereas only six DEGs were observed between NoAdditive- and TGFβ3-treated cells, indicating greater transcriptional similarity between the latter two conditions. Consistently, heatmap analysis showed two opposing transcriptional clusters separating SB431542- and TGFβ3-treated iEPDCs, while NoAdditive-treated cells occupied an intermediate position. Together, these findings indicate that TGFβ pathway modulation induces gradual transcriptional remodeling rather than a strictly binary response, consistent with the concept of EMT as a graded and dynamic process in developmental and injury-associated contexts [6,44].

Comparison of SB431542-treated and TGFβ3-treated iEPDCs revealed differential expression of several transcripts associated with epithelial and epicardial identity. SB431542-treated iEPDCs exhibited elevated expression of *ITLN1*, *MSLN*, *PRG4*, *HP*, *CRIP1* and *UPK3B*, several of which have been previously associated with mesothelial and epicardial cell states in fetal and adult human epicardium [27,32,45]. *TXNIP* was also increased under SB431542. Since *TXNIP* has previously been linked to hypoxia-associated cellular responses in primary EPDCs [46], its elevated expression may represent an additional transcript associated with the epithelial-like iEPDC state. Another gene upregulated in SB431542-treated iEPDCs was *PTGDS*. As PTGDS encodes a secreted prostaglandin D synthase with reported anti-inflammatory and cardioprotective properties [47], its increased expression may point to paracrine functions associated with the epithelial-like state.

In contrast, comparison of TGFβ3-treated and SB431542-treated iEPDCs revealed increased expression of several mesenchymal and remodeling-associated transcripts in TGFβ3-treated cells, including *FN1*, *COL1A1*, *SERPINE1*, *TGFB2* and *LTBP2*.

*FN1* and *COL1A1* encode ECM components, and their upregulation supports activation of an ECM-remodeling program in TGFβ3-treated iEPDCs. This is in line with previous observations in TGFβ3-treated human EPDCs and inducible proliferative EPDCs [17], while our RNAseq data extend these findings by revealing a broader treatment-dependent remodeling signature in adult human iEPDCs. Moreover, *TGFB2, TGFB1* and *LTBP2* were upregulated in TGFβ3-treated iEPDCs compared to SB431542-treated cells. This may indicate activation of a broader TGFβ signaling program beyond the initial exogenous stimulus. TGFβRI/II–SMAD2/3 signaling has also been implicated in serum-induced proliferation of hCSCs [48]. While *TGFB2* upregulation could suggest a potential autocrine reinforcement of TGFβ signaling [49], increased *LTBP2* expression may reflect enhanced extracellular matrix remodeling and altered TGFβ bioavailability, both processes commonly associated with EMT [49,50]. In addition, TGFβ3-treated iEPDCs showed elevated *SERPINE1* expression, a TGFβ-responsive gene associated with extracellular remodeling and EMT-related programs [51,52]. Interestingly, increased *SERPINE1* expression has also previously been reported in EPDCs compared with iPSC-derived cardiac progenitor cells (CPCs) [46]. Beyond these remodeling-associated transcripts, TGFβ3-treated iEPDCs also showed increased expression of *IL11*, *HBEGF*, *VEGFC* and *TNC*. These genes have been previously linked to cardiac remodeling, post-myocardial infarction remodeling, growth factor signaling, lymphangiogenesis-mediated inflammation resolution, and injury-associated ECM remodeling in rat and mouse models [53,54,55,56], which supports the activation of a broader transcription program associated with injury responses and tissue remodeling in TGFβ3-treated iEPDCs. Further, *ATF6B* was upregulated in TGFβ3-treated iEPDCs compared with SB431542-treated cells. *ATF6B* encodes activating transcription factor 6 beta, a component of the unfolded protein response [57]. Although its role in human EPDCs remains unclear, ATF6-family signaling has been implicated in adaptation to cardiac stress and remodeling in mouse models [58,59]. ATF6 activation has been linked to cardiac hypertrophic growth and proteostasis during pressure overload-induced remodeling [59,60]. In particular, ATF6α and ATF6β have been shown to regulate partially overlapping cardiac gene programs and to influence pressure overload-induced hypertrophic remodeling in mouse hearts [58]. In human cardiac stem cells, it was identified as a potential NF-κB target involved in serum-induced proliferation [61]. Therefore, *ATF6B* upregulation in TGFβ3-treated iEPDCs may be linked to stress- and remodeling-associated transcriptional responses accompanying mesenchymal-like activation.

Interestingly, NoAdditive-treated iEPDCs displayed an intermediate transcriptional profile between SB431542- and TGFβ3-treated cells, but were overall more similar to the TGFβ3-treated condition. Whereas the comparison of NoAdditive- and TGFβ3-treated iEPDCs revealed six DEGs, substantially larger transcriptional differences were observed between SB431542- and NoAdditive-treated cells. This indicates that the NoAdditive culture condition was transcriptionally distinct from the epithelial-like state induced by TGFβ pathway inhibition. However, NoAdditive-treated iEPDCs retained higher expression of epithelial-associated junction transcript *TJP1_2* compared with TGFβ3-treated cells. Together, these findings suggest that NoAdditive-treated iEPDCs do not represent a stable epithelial-like state but rather an intermediate activation state with partial retention of epithelial-associated features.

Comparison of the selected marker transcripts with the ICC findings revealed broadly similar treatment-associated patterns for *CRIP1* and *MSLN*. *WT1* showed less pronounced differences at the transcript level, while *VIM* and *ENG* exhibited variable but generally corresponding trends. Together, the RNAseq and protein-level data provide complementary evidence for treatment-associated phenotypic changes.

To further examine whether these phenotypic changes were accompanied by a broader EMT-associated transcriptional response, additional EMT- and remodeling-associated genes were analyzed. The tendency toward higher transcript abundance of the EMT-associated factors *SNAI1*, *SNAI2* and *TWIST1*, together with *CDH2* and the matrix-remodeling genes *MMP2* and *MMP9*, provides additional transcriptional support for the EMT-associated phenotypic and migratory changes observed following TGFβ3 treatment [7,62]. These findings complement observed changes in cell morphology and epithelial- and mesenchymal-associated protein expression.

Beyond these treatment-associated changes, selected genes associated with epicardial and cardiac cell identity were examined to further characterize the iEPDC populations. Across all three treatment conditions, *TCF21* and *TBX18* showed higher abundance than *GATA4* and *TNNT2*. *TCF21* and *TBX18* are associated with epicardial and EPDC identity across species and have been detected at the protein level in fetal and adult primary EPDCs [24,63,64], consistent with the epicardial origin of the iEPDC populations.

Moreover, GATA4, a cardiac transcription factor involved in heart development and in injury-responsive cardiac processes, and the cardiomyocyte-associated marker TNNT2 were detected at comparatively low transcript levels and showed no clear treatment-associated pattern [33,65]. The low *GATA4* expression may be related to the atrial origin of the iEPDCs, but this would require direct comparison with ventricular EPDCs. Overall, these findings suggest that TGFβ signaling primarily affected the epithelial-like and mesenchymal-like state of the iEPDCs rather than altering the expression of these selected cardiac-associated genes.

KEGG pathway enrichment analysis further supported the existence of two opposing transcriptional programs corresponding to epithelial-like and mesenchymal-like states. In SB431542-treated iEPDCs, enrichment of the ‘Rap1 signaling pathway’ compared to TGFβ3-treated cells is consistent with the maintenance of epithelial-like integrity. Rap1 has been shown to regulate E-cadherin-mediated cell–cell adhesion [66,67] and to promote the formation of E-cadherin-based adherence junctions [68]; processes that are central to epithelial homeostasis. Thus, enrichment of Rap1 signaling in SB431542-treated cells supports stabilization of an epithelial-like phenotype. In contrast, TGFβ3-treated iEPDCs exhibited enrichment of pathways associated with mesenchymal transition. The increased representation of the ‘PI3K-Akt signaling pathway’ has been linked to EMT [69,70]. Enrichment of integrin signaling further supports acquisition of mesenchymal-like features, consistent with altered cell–matrix interaction during partial EMT-associated remodeling [71,72]. For instance, αvβ3 integrin has been reported to induce partial EMT independently of TGFβ signaling [71], and transcriptional activation of integrin β6 has been described during EMT [72], reflecting altered cell–matrix interactions. Enrichment of ‘focal adhesion’ and ‘regulation of actin cytoskeleton’ pathways is consistent with processes associated with migratory behavior and structural remodeling during EMT [73,74].

Consistently, GO term analysis demonstrated enrichment of biological processes related to ‘extracellular matrix organization’, cell adhesion and cytoskeletal remodeling in TGFβ3-treated iEPDCs, consistent with the observed morphological changes. Together, these KEGG and GO terms data indicate that TGFβ modulation was associated with shifts in iEPDCs into opposing epithelial-like and mesenchymal-associated programs. In line with recent work by Oliveira and colleagues highlighting the complexity of epicardial secretome in murine embryonic epicardial-derived cell line [75], these findings support the use of this model to investigate differences in extracellular matrix remodeling and potential paracrine signaling between epithelial-like and mesenchymal-like iEPDC states.

Interestingly, biological process enrichment in SB431542-treated iEPDCs compared to TGFβ3-treated cells included ion-binding-related and zinc-binding-related terms, which may in part relate to elevated expression of several metallothionein genes. CRIP1 belongs to the LIM/double-zinc finger protein family [76] and has previously been proposed as an epithelial EPDC marker and potential regulator of EMT-related processes [27]. Here, we studied for the first time the expression of CRIP1 in adult human iEPDCs in vitro.

Elevated CRIP1 expression in SB431542-treated iEPDCs compared to TGFβ3-treated cells was consistently observed at both transcriptional and protein levels, supporting its association with epithelial-like epicardial characteristics. While the precise functional role of CRIP1 in epicardial activation remains incompletely understood, these findings suggest that CRIP1 may represent a useful marker for investigating epithelial-like epicardial states and EMT-related plasticity.

Finally, scratch assay experiments demonstrated enhanced migratory behavior in TGFβ3-treated iEPDCs compared with SB431542-treated cells, functionally supporting the mesenchymal-like cellular characteristics observed following TGFβ3 treatment. This functional response is consistent with enriched ‘wound healing’ and ‘extracellular matrix organization’ GO terms in TGFβ3-treated iEPDCs and supports the activation of a remodeling-associated phenotype.

Taken together, the combined morphological, protein-level, transcriptomic and migratory findings indicate that the iEPDCs retained TGFβ-responsive phenotypic plasticity following immortalization. Rather than demonstrating a complete EMT, the data support a treatment-dependent shift along an epithelial-like to mesenchymal-like spectrum, with SB431542-treated iEPDCs displaying the most pronounced epithelial-associated features, TGFβ3-treated cells showing enhanced mesenchymal- and remodeling-associated characteristics. NoAdditive-treated cells occupied an intermediate position but were transcriptionally more similar to the TGFβ3-treated condition than to cells under TGFβ pathway inhibition.

The TGFβ3-associated morphological, molecular and migratory changes observed in the present study are broadly consistent with those reported by Ge and colleagues, who established an inducible proliferative adult human EPDC model [17]. All five donor-derived iEPDC populations included in the present study expressed the epicardial- and epithelial-associated markers WT1, MSLN and CRIP1. Building on this initial characterization, treatment-dependent changes in WT1, MSLN, CRIP1, VIM and CD105 were quantitatively assessed at the protein level in three independent donor-derived populations and complemented by protein-coding transcriptomic analysis across all five populations. The present study, therefore, extends previous work by combining an expanded marker panel with protein-coding transcriptomic characterization of epithelial-like and mesenchymal-like iEPDC states. Based on more recent evidence identifying CRIP1 in human fetal and adult epicardial tissue and primary fetal EPDCs, we additionally characterized its treatment-dependent expression at the protein and transcript level in immortalized adult human EPDCs [27].

The treatment-dependent responses observed in this study suggest that iEPDCs may provide a human in vitro model for investigating defined aspects of injury-associated epicardial activation and plasticity [16]. In addition to TGFβ pathway modulation as one application, the model could be used to study responses to disease-associated stimuli. In future studies, secretome and co-culture analyses could further determine whether changes in iEPDC state influence epicardial–myocardial crosstalk, thereby extending the model toward more disease-relevant experimental settings [75,77].

One limitation of this study is that SV40LT-mediated immortalization may influence transcriptional programs and cellular properties compared with primary adult EPDCs. Nevertheless, the iEPDCs retained selected epicardial-associated characteristics and exhibited treatment-associated responses to TGFβ3 and SB431542 at the transcript, protein, and functional levels.

The iEPDCs used in this study were derived from human atrial epicardial tissue. Given the distinct molecular profiles of human atrial and ventricular cardiac regions [78,79], findings from atrial-derived iEPDCs cannot be directly extrapolated to ventricular epicardial biology, particularly because myocardial infarction predominantly affects the ventricular myocardium, especially the left ventricle [80,81]. Future studies should directly compare atrial and ventricular EPDCs to determine whether regional origin influences their basal phenotype, functional properties, and responses to disease-relevant stimuli. Such comparisons remain challenging because access to viable ventricular epicardial tissue is limited. Nevertheless, the atrial-derived iEPDCs displayed an epicardial-associated marker profile and responded to TGFβ pathway modulation with epithelial- and mesenchymal-like phenotypic changes, supporting their use as a human in vitro model of epicardial plasticity.

## 5. Conclusions

In summary, adult human iEPDCs retained epicardial- and epithelial-associated characteristics following immortalization and provide an expandable in vitro model to study TGFβ pathway-dependent epicardial activation. By integrating phenotypic, transcriptomic and functional analyses, this study demonstrates coordinated treatment-dependent shifts between epithelial-like and mesenchymal-like states. SB431542 supported epithelial-like characteristics, whereas TGFβ3 induced changes consistent with an EMT-associated cellular response. Furthermore, the treatment-dependent regulation of CRIP1 at both the transcript and protein levels supports its potential as a marker of an epithelial-like state in adult human iEPDCs. Together, these findings support the use of iEPDCs as a human cell-based model for investigating adult epicardial plasticity and injury-associated remodeling.

## Figures and Tables

**Table 1:** Primary antibodies used for ICC.

Antibody	Manufacturer	Dilution
mouse anti-WT1	6F-H2, Invitrogen, Carlsbad, CA, USA	1:100
mouse anti-SV40LT	SC-147, Santa Cruz Biotechnology, Heidelberg, Germany	1:400
rabbit anti-CRIP1	# PA5-24643, Invitrogen, Carlsbad, CA, USA	1:50
rabbit anti-Mesothelin	# PA5-79697, Invitrogen, Carlsbad, CA, USA	1:500
Alexa Fluor™ 488 Phalloidin	A12379, Invitrogen, Carlsbad, CA, USA	1:40
goat anti-Endoglin/CD105	AF1097, R&D Systems, Minneapolis, MN, USA	1:50
rabbit anti-Vimentin	D21H3, Cell Signaling, Danvers, MA, USA	1:200

## Data Availability

The original RNAseq raw data of iEPDCs generated in this study are openly available in the NCBI Gene Expression Omnibus under the accession number GSE330909. Publicly available RNAseq datasets analyzed in this study were obtained from NCBI GEO under accession numbers GSE129547 and GSE301005.

## Supplementary Materials

The following supporting information can be downloaded at: https://www.mdpi.com/article/10.3390/cells15141313/s1. Figure S1: Characterization of marker retention, SV40LT expression, and proliferative properties of iEPDCs. Figure S2: RNAseq-derived transcript per million (TPM) values of selected ICC-associated markers in iEPDCs cultured under SB431542, NoAdditive and TGFβ3 conditions for five days. Figure S3: RNA-seq-derived transcripts per million (TPM) values of selected markers in iEPDCs cultured for 5 days under SB431542, NoAdditive, or TGFβ3 conditions; Figure S4: Heatmap of the top 200 differentially expressed genes with all gene names displayed in iEPDCs following 5 days of treatment with SB431542, NoAdditive, or TGFβ3; Table S1: List_DEGs_iEPDCs_TGFB3vsiEPDCs_SB431542; Table S2: List_DEGs_iEPDCs_SB431542vsiEPDCs_NoAdditive; Table S3: List_DEGs_iEPDCs_TGFB3vsiEPDCs_NoAdditive.

## Abbreviations

The following abbreviations are used in this manuscript:

ATF6B	Activating transcription factor 6 beta
CD105	Cluster of differentiation 105/Endoglin
COL1A1	Collagen type I alpha 1 chain
CRIP1	Cysteine-rich protein 1
DEG	Differentially expressed gene
ECM	Extracellular matrix
EMT	Epithelial-to-mesenchymal transition
EPDC	Epicardium-derived cell
FCS	Fetal calf serum
FN1	Fibronectin 1
GEO	Gene Expression Omnibus
GO	Gene Ontology
hCSC	Human cardiac stem cell
HP	Haptoglobin
hPSC-EPC	Human pluripotent stem cell-derived epicardial cell
ICC	Immunocytochemistry
iEPDC	Immortalized epicardium-derived cell
ITLN1	Intelectin 1
KEGG	Kyoto Encyclopedia of Genes and Genomes
LTBP2	Latent transforming growth factor beta binding protein 2
MSLN	Mesothelin
PBS	Phosphate-buffered saline
PCA	Principal component analysis
PFA	Paraformaldehyde
PRG4	Proteoglycan 4
PTGDS	Prostaglandin D2 synthase
RNAseq	RNA sequencing
SV40LT	Simian virus 40 large T antigen
TGFB1	Transforming growth factor beta 1
TGFB2	Transforming growth factor beta 2
TGFβ	Transforming growth factor beta
TGFβRI	Transforming growth factor beta receptor I
TJP1	Tight junction protein 1
TNFα	Tumor necrosis factor alpha
TPM	Transcripts per million
TXNIP	Thioredoxin-interacting protein
UPK3B	Uroplakin 3B
VEGFC	Vascular endothelial growth factor C
VIM	Vimentin
WT1	Wilms’ tumor 1
