# Supplementary material for: TGFβ-Dependent Epithelial–Mesenchymal Plasticity in Immortalized Human Atrial Epicardial Cells: An mRNA Profiling Study
Source: Cells. 2026 Jul 22;15(14):1313. doi: 10.3390/cells15141313 (PMC13406294; doi:10.3390/cells15141313)
Supplement: Supplementary file 1 [file cells-15-01313-s001.zip › Supplements_Figures.pdf]

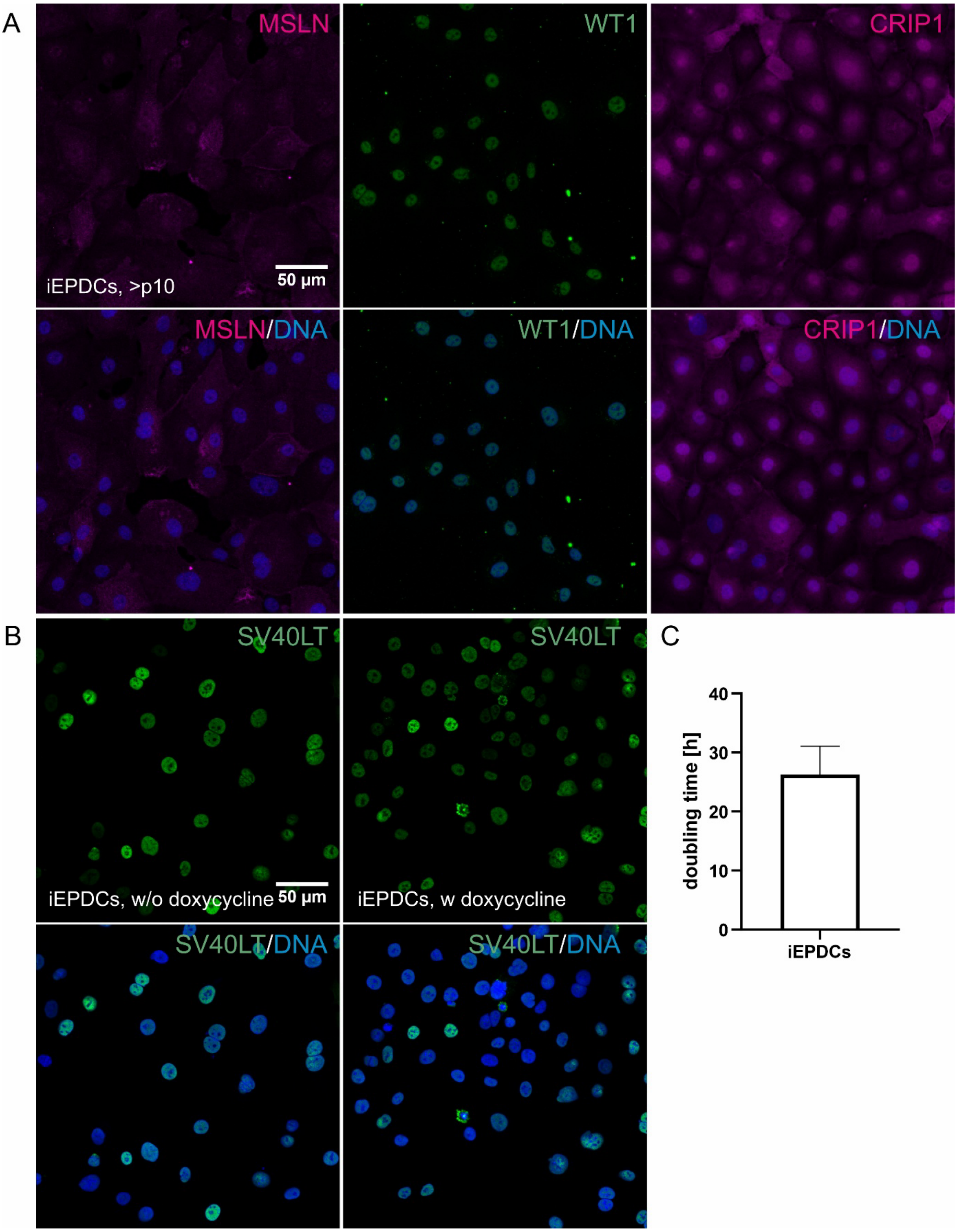

Figure S1. Characterization of marker retention, SV40LT expression, and proliferative properties of iEPDCs. (A) Immunocytochemical analysis of human iEPDCs showing preserved expression of the epithelial epicardial-associated markers MSLN, WT1, and CRIP1 at higher passage (P14). (B) Immunocytochemical analysis of SV40LT expression in iEPDCs cultured with (w/) or without (w/o) doxycycline for 14 days. (C) Population doubling time of human iEPDCs derived from three independent donors. Data are shown as mean ± SD ((n=3) donors).

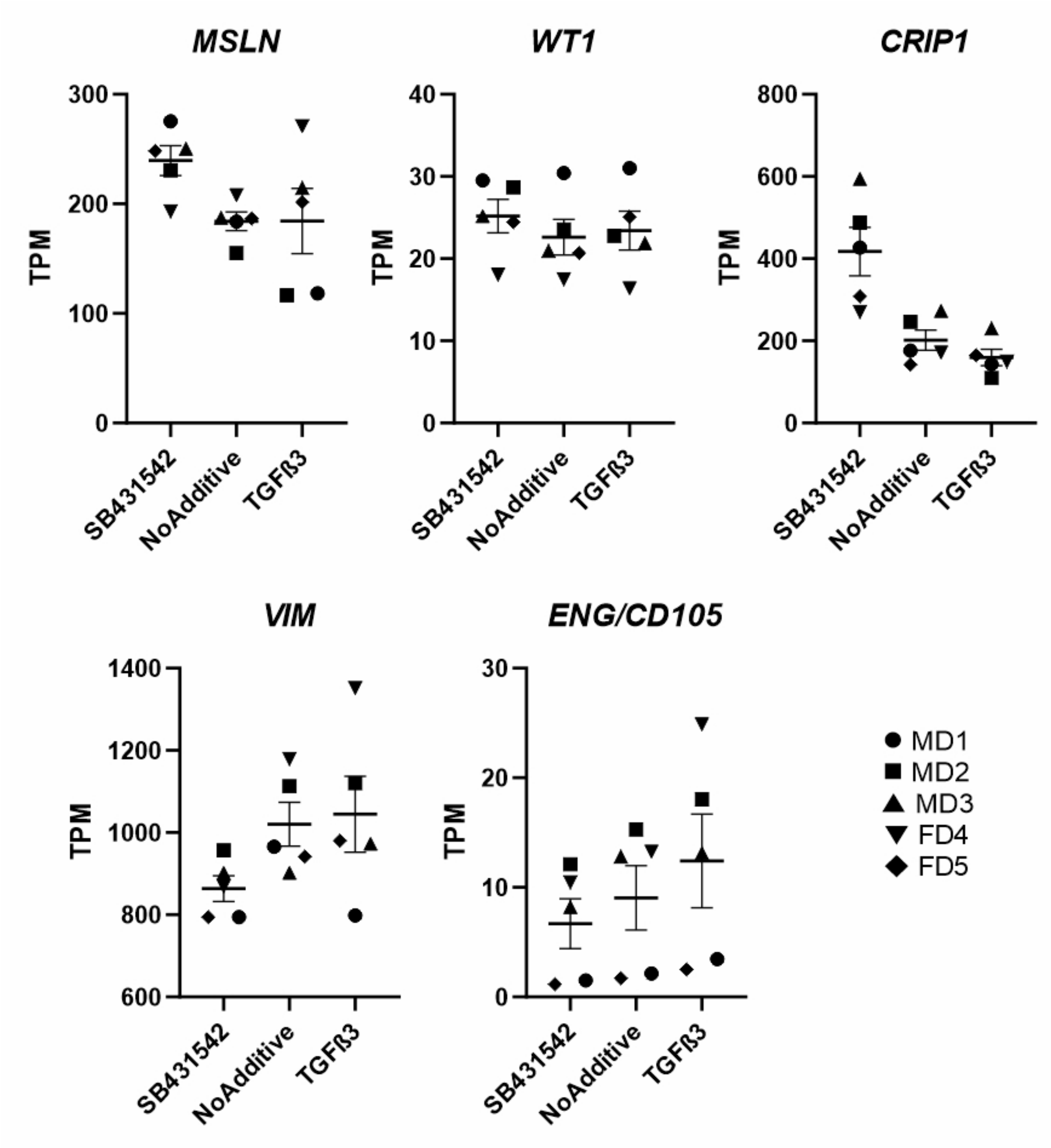

Figure S2 RNAseq-derived transcript per millions (TPM) values of selected ICC-associated markers in iEPDCs cultured under SB431542, NoAdditive and TGFβ3 conditions for five days. Scatter plots show transcript abundance of epithelial-like epicardial-associated markers (*WT1*, *MSLN* and *CRIP*1) and activation-associated markers (*ENG* and *VIM*) across individual donors. RNAseq-derived transcript abundance showed treatment-associated trends partly resembled to the observed the observed immunocytochemical patterns despite donor-dependent variability. All data are shown as mean ± SEM.

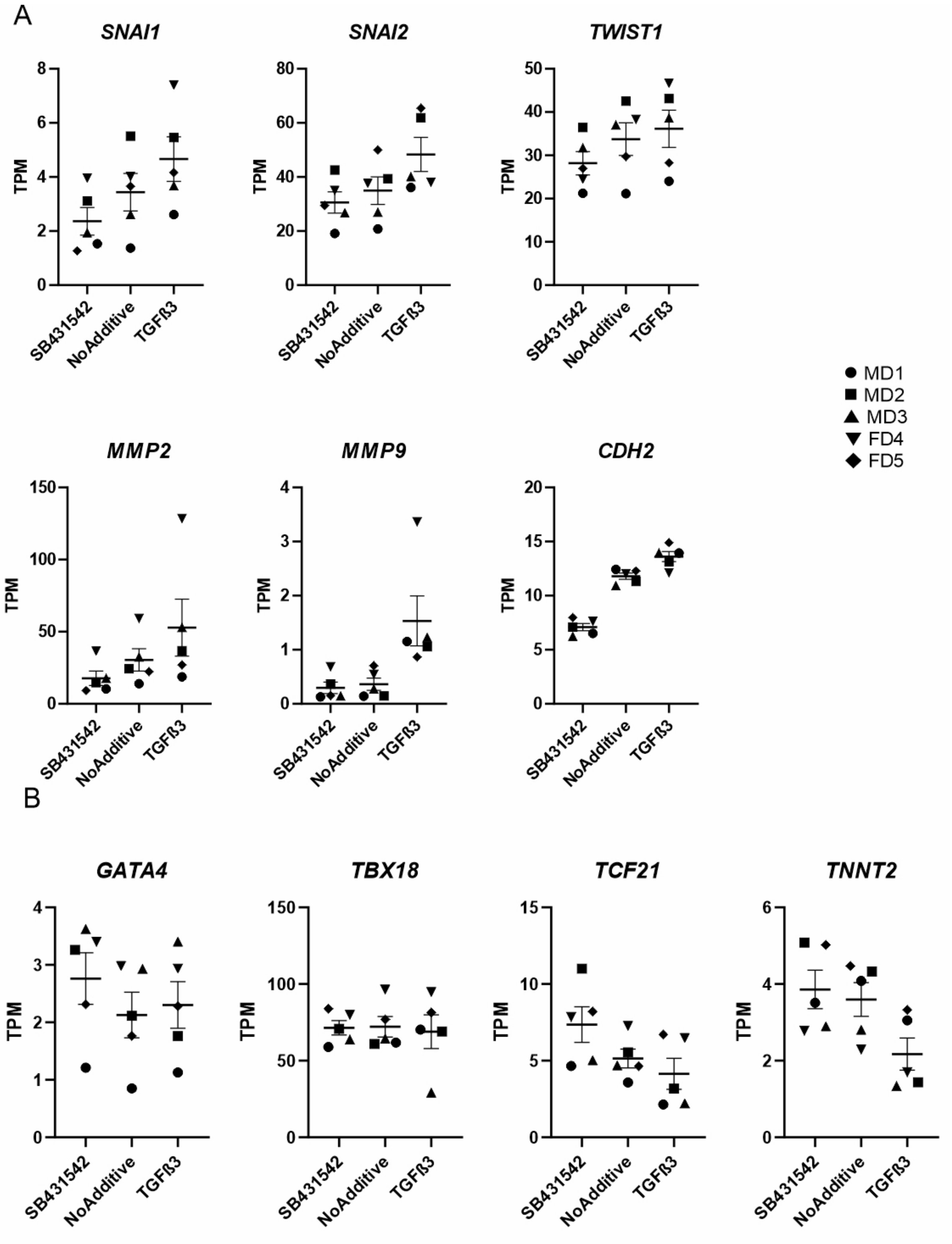

Figure S3 RNA-seq-derived transcripts per million (TPM) values of selected markers in iEPDCs cultured for 5 days under SB431542, NoAdditive, or TGFβ3 conditions. (A) EMT- and ECM-remodeling-associated markers *SNAI1*, *SNAI2*, *TWIST1*, *MMP2*, *MMP9*, and *CDH2*. (B) Epicardial- and cardiac-associated markers *GATA4*, *TBX18*, *TCF21*, and *TNNT2*. Data are presented as mean ± SEM.

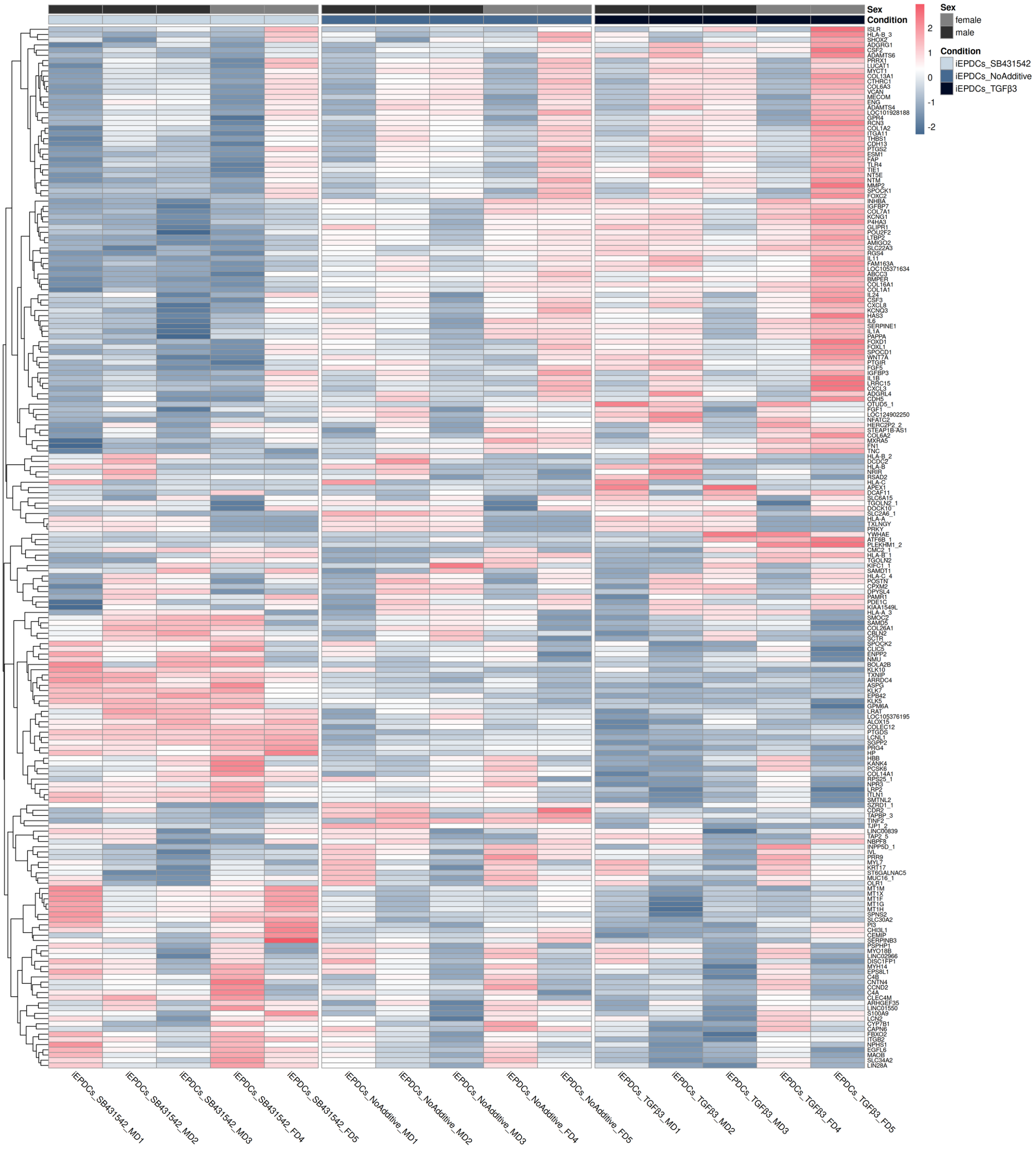

Figure S4 Heatmap of the top 200 differentially expressed genes with all gene names displayed in iEPDCs following 5 days of treatment with SB431542, NoAdditive, or TGFβ3. The complete labeling facilitates detailed visualization of treatment-associated expression patterns.
